# The seasonal development dynamics of the yak hair cycle transcriptome

**DOI:** 10.1186/s12864-020-6725-7

**Published:** 2020-05-11

**Authors:** Pengjia Bao, Jiayu Luo, Yanbin Liu, Min Chu, Qingmiao Ren, Xian Guo, Bolin Tang, Xuezhi Ding, Qiang Qiu, Heping Pan, Kun Wang, Ping Yan

**Affiliations:** 1 Lanzhou Institute of Husbandry and Pharmaceutical Sciences, Chinese Academy of Agricultural Sciences, Lanzhou, 730050 China; 2 Key Laboratory for Yak Breeding Engineering of Gansu Province, Lanzhou, 730050 China; 3 State Key Laboratory of Grassland Agro-Ecosystem, School of Life Sciences, Lanzhou University, Lanzhou, 730000 China; 4 School of Ecology and Environment, Northwestern Polytechnical University, Xi’an, 710072 China; 5 Northwest Minzu University Life Science and Engineering College, Lanzhou, 730030 China

**Keywords:** Hair cycle, Seasonal development, Transcriptome, Yak

## Abstract

**Background:**

Mammalian hair play an important role in mammals’ ability to adapt to changing climatic environments. The seasonal circulation of yak hair helps them adapt to high altitude but the regulation mechanisms of the proliferation and differentiation of hair follicles (HFs) cells during development are still unknown. Here, using time series data for transcriptome and hormone contents, we systematically analyzed the mechanism regulating the periodic expression of hair development in the yak and reviewed how different combinations of genetic pathways regulate HFs development and cycling.

**Results:**

This study used high-throughput RNA sequencing to provide a detailed description of global gene expression in 15 samples from five developmental time points during the yak hair cycle. According to clustering analysis, we found that these 15 samples could be significantly grouped into three phases, which represent different developmental periods in the hair cycle. A total of 2316 genes were identified in these three consecutive developmental periods and their expression patterns could be divided into 9 clusters. In the anagen, genes involved in activating hair follicle growth are highly expressed, such as the *WNT* pathway, *FGF* pathway, and some genes related to hair follicle differentiation. In the catagen, genes that inhibit differentiation and promote hair follicle cell apoptosis are highly expressed, such as *BMP4*, and *Wise*. In the telogen, genes that inhibit hair follicle activity are highly expressed, such as *DKK1* and *BMP1*. Through co-expression analysis, we revealed a number of modular hub genes highly associated with hormones, such as *SLF2, BOP1 and DPP8.* They may play unique roles in hormonal regulation of events associated with the hair cycle.

**Conclusions:**

Our results revealed the expression pattern and molecular mechanisms of the seasonal hair cycle in the yak. The findings will be valuable in further understanding the alpine adaptation mechanism in the yak, which is important in order to make full use of yak hair resources and promote the economic development of pastoral plateau areas.

## Background

Since the Cenozoic era, mammals have become among the dominant species on earth, adapting to a variety of ecological niches such as plateaus, deserts and even oceans. The diversity and periodic regulation of mammalian hair play an important role in mammals’ ability to adapt to varying climatic environments [1]. Mammalian hair is a highly keratinized tissue produced by hair follicles (HFs) within the skin. There are two kinds of hair follicles: the primary HFs produces the coarse coat hair in all mammals, and the secondary HFs can produce the cashmere or ‘fine hair’ of certain mammals, including yak [2]. After maturation, the hair follicles begin to grow periodically throughout life. The periodic expression of HFs growth is characterized by a change in HFs morphology and HFs gene expression over time. The hair cycle can be divided into a growth phase (anagen), a degenerative period (catagen) and a rest period (telogen) [3]. The HFs cycle maintains its normal periodicity in response to a large and complex network of signaling pathways, which are differentially expressed in time and space to regulate hair follicle growth. Over the past few decades, signals that control the proliferation and differentiation of HFs cells during development and homeostasis have been studied extensively. Several transcription factors, such as *Tcf3/4*, *Lhx2*, *p63*, *Dlx3*, *Lef1*, *Msx2/Foxn1*, etc., play critical roles in hair follicle stem cell activation, self-renewal, differentiation, and cycling by modulating several key signaling pathways [4].

The yak (*Bos grunniens*), which is a key species in the Qinghai-Tibet Plateau (QTP), is a rare large animal; it is a Chinese endemic species that lives in arctic-alpine and hypoxic environments [5]. For thousands of years, yaks have played an essential role in maintaining the survival of the Tibetan people, providing local herdsmen with essential means of production and livelihood, such as clothing, food, shelter and transportation. The yak’s coat is strongly influenced by the lifestyle of Tibetan people. In June, when the weather is warm, the pastoralists collect the yak’s hair and exploit it for economic benefit and living materials. Compared with other, lower-altitude cattle species, the development of yak villus and hair follicles has very significantly different characteristics; this is an important factor in the ability of the yak to adapt to the extreme environment of the plateau, and also makes yak an excellent model species for the study of hair development. Firstly, the chest, legs and flanks of the yak have long, thick skirt hair, which traps air; it is thicker than the air layer provided by a single type of hair and forms a natural thermal insulation layer. In addition, the yak responds very sensitively to the different seasons.

The hair of yak experienced seasonal development circle, like many other mammals and birds [6, 7], and is cycling once a year, with or without human intervention [8]. Previous studies divided each year’s cycle into three phases, also named as anagen, catagen and telogen. In the anagen, the number of primary HFs and secondary HFs increases obviously. Then in the catagen, the number of HFs decreased, and the secondary HFs shrink. At the telogen, the HFs group structure is more loose and the hair start fall off. Both the primary and secondary HFs experienced periodic cycle [9] but the increase of secondary HFs in cold season were thought to be the main basis for the yak’s resistance to the cold and harsh highland environment [10]. This seasonal cycling allows yak to have thicker hair in cold winters and sparser hair in warmer seasons [7]. Previous studies had found many genes and pathways that are involved in the formation and growth of the yak hair follicle, but a systematically analysis is still lacked [11–13].

The purpose of our study was to identify the regulatory pathways and key genes related to the periodic changes undergone by yak hair follicles in a large scale investigation by collecting yak skin samples during different seasons for comprehensive trait testing, hormone content determination and transcriptome sequencing; Using the resulting data, we analyzed the interaction of these genes, established a regulatory network of related genes, and investigated in depth the mechanism by which hair follicle development is regulated in the yak. We believe that this study will not only provide valuable reference information for insight into the molecular mechanism of animal hair follicle development, but also serve as the basis for breeding new strains of yak and developing and utilizing hair resources.

## Results

### Overview of RNA-seq data

A total of 15 libraries were sequenced from skin tissues of yaks at five different physiological stages (*n* = 3 for each). We selected these particular developmental time points because they could cover the three phases and are at the key turning points in the year when the temperature changes dramatically, and such temperature changes are one of the important factors affecting the cyclical changes in yak coats. (Additional file 1: Figure S1). The average amount of raw data for these 15 libraries was 25.15 million reads with 3.94 Gb of raw bases. After discarding low-quality reads, an average of 24.96 million clean reads was obtained, an amount of data sufficient to meet the requirements of this research. The clean reads were mapped to the yak reference genome using Hisat2 software, and the percentage of the total reads mapped was approximately 92.21% for each sample, indicated that the yak reference genome we used was appropriate. Specifically, 91.94, 92.63, 92.59, 91.86 and 92.04% of the clean reads from RNA sampled in Aug, Oct, Jan, Mar and Jun respectively were mapped to the yak reference genome (Additional file 2: Table S1).

**Fig. 1:**
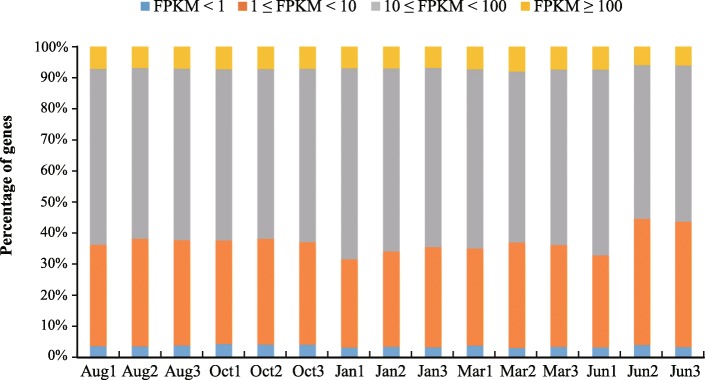
Transcript expression levels in the fifteen samples

We used StringTie software to produce more complete and accurate reconstructions of genes and better estimates of expression levels. A gene was considered to be expressed in a given sample if the lower boundary of its FPKM 95% confidence interval was greater than zero [14]. Based on this criterion, 11,666 genes were identified as being expressed in at least one of the 15 samples, and the distribution of FPKM values for these genes was then statistically analyzed. The results showed that 90% of the genes were expressed at a medium level (1 ≤ FPKM < 100), less than 8% exhibited high-level expression (FPKM ≥100) and the rest showed low-level expression (FPKM < 1) among the 15 samples analyzed. We found that the proportions of genes at the three expression levels (high, medium, and low) were similar in all samples, indicating that our transcriptome data were consistent and lacked any obvious bias (Fig. 1).

### Hierarchical clustering and principal component analysis of samples

To assess the consistency of sample collection and explore the transcriptomic relationships among the five stages, we performed hierarchical clustering and principal component analysis (PCA) for these samples using the whole gene expression dataset.

**Fig. 2:**
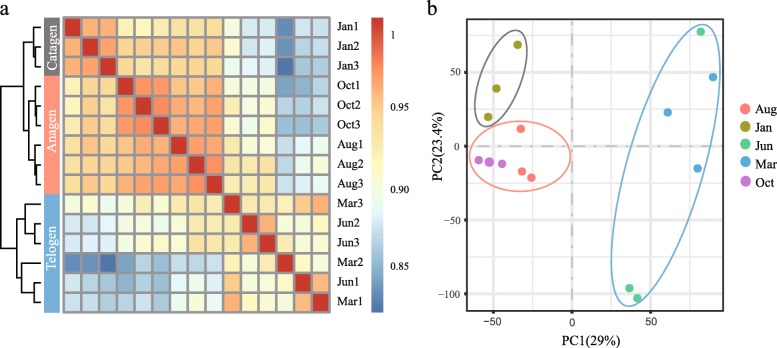
Sample clustering for yaks at different stages in the hair cycle. **a** Heat map and hierarchical clustering of samples from five stages; **b** PCA of samples from five stages

Using hierarchical clustering, we found that the samples were divided into three clusters and that samples from the same period were grouped together, with the Jan samples forming a single cluster, the Aug and Oct samples grouped together and the Mar and Jun samples being another cluster (Fig. 2a). In the PCA analysis, the samples collected in the same period were grouped into the same cluster; this is consistent with the results of hierarchical clustering (Fig. 2b). Both results showed that the 15 samples could be grouped into three significant phases, and we speculated that these three stages might represent different developmental components of the yak hair cycle. The hair cycle is usually divided into three phases: anagen (growth phase), catagen (regression phase) and telogen (resting phase). Based on the period when yak hair growth is known to occur and on records of mean monthly temperatures, we hypothesized that the Aug and Oct samples were in the anagen of the hair cycle, the Jan samples were in the catagen and the Mar and Jun samples were in the telogen.

### Change in transcriptome profiles during the hair cycle

**Fig. 3:**
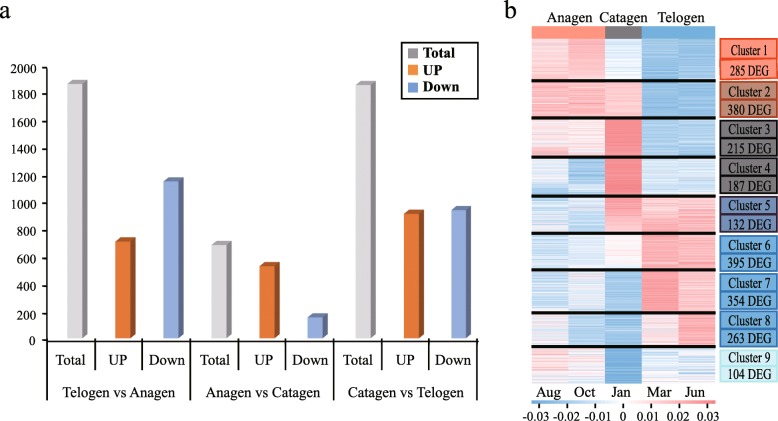
Differential gene expression among the anagen, catagen, and telogen of the hair cycle. **a** Numbers of genes differentially expressed between the libraries compared. Total DEGs (grey), up-regulated genes (orange), and down-regulated genes (blue) are shown as a histogram; **b** Hierarchical clustering and heat map for all samples based on differential gene expression data

In this study, we used DESeq2 software to identify the genes that were differentially expressed (DEGs) between consecutive stages to determine changes during the hair cycle, resulting in the identification of 2316 DEGs. Of these DEGs, 714, 534 and 916 genes were upregulated and 1153, 155 and 943 genes were downregulated in telogen versus anagen, anagen versus catagen, catagen versus telogen respectively (Fig. 3a). It was clear that, compared to anagen versus catagen, a larger number of genes was up- or down-regulated in telogen versus anagen, and catagen versus telogen, indicating a major change in the transcriptional profile (Additional file 3: Figure S2).

The expression patterns make it clear that there are multitudinous and complex interactions among genes, and genes with similar expression patterns may have similar functions in the hair cycle. An in-depth study of gene expression patterns during the hair cycle may therefore help in gaining a better understanding of the mechanism regulating this cycle. We further investigated the expression patterns of DEGs at three developmental stages during the hair cycle. We classified these 2316 DEGs into nine gene clusters (C1-C9) based on their expression patterns (Fig. 3b), Each cluster represents a unique set of expression patterns.(1) Cluster one: each gene is upregulated in the anagen and downregulated in the other two periods; (2) Cluster two: continuous upregulation from anagen to catagen, but downregulated in telogen; (3) Cluster three: upregulated from anagen to catagen and downregulated in telogen, but mainly expressed in the catagen; (4) Cluster four: upregulated in the catagen and downregulated in the other two periods; (5) Cluster five: downregulated in the anagen and upregulated from catagen to telogen; (6) Clusters six and seven: upregulated during the telogen and downregulated in the other two periods; (7) Cluster eight: upregulated during the latter part of the telogen; (8) Cluster nine: downregulated during the catagen. We found the largest number of DEGs in Cluster 6 (upregulated during the telogen), which included 395 genes (17.1%), and the second largest number was in Cluster 2 (downregulated in the telogen) with 380 (16.4%).

### GO and pathway analysis of DEGs

**Fig. 4:**
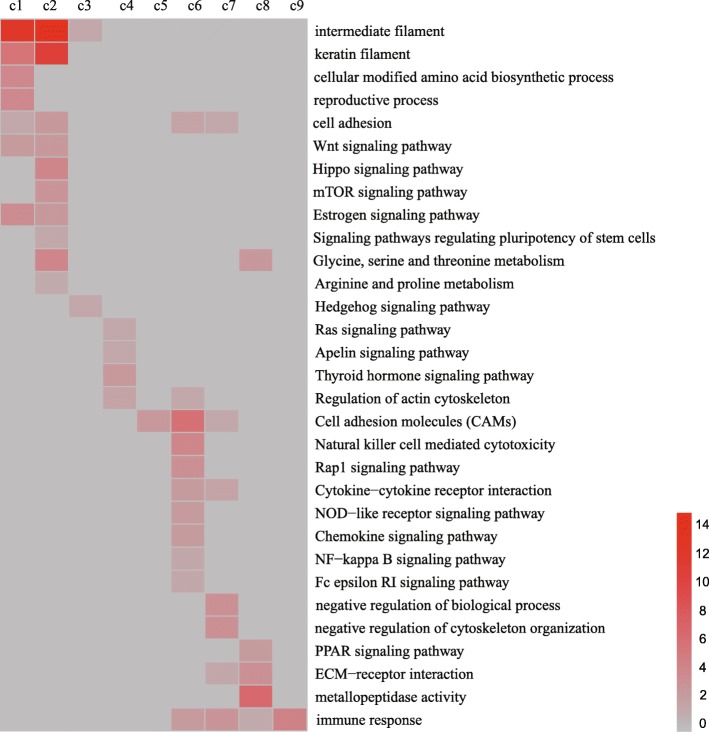
Functional analysis of genes in the nine clusters of DEGs. The intensity of the red coloration indicates the magnitude of the log of the value of P for importance. Gray represents missing values

Significant functional categories (*p* < 0.05) differentially expressed during the yak hair cycle were identified in GO and pathway analysis applied to the DEGs falling into the nine clusters, and 84 GO terms (Additional file 4: Table S2) and 147 KEGG pathways (Additional file 5: Table S3) were enriched with a corrected *P*-value less than 0.05. The results showed enrichment for particular clusters of gene expression (Fig. 4). For example, genes involved in the synthesis of intermediate filament and keratin filament, the Wnt signaling pathway, signaling pathways regulating the pluripotency of stem cells, the Hippo signaling pathway and the MTOR and Hedgehog signaling pathways are greatly enriched among the genes that show peak expression in the anagen and catagen (Clusters C1, C2 and C3). The results presented above suggest that the GO terms and KEGG pathway of genes highly expressed in the anagen and catagen are related to signal induction and hair generation. Genes showing peak expression in the telogen (clusters C6 and C7) included some that are required for Natural killer cell mediated cytotoxicity and negative regulation of biological process, the Nature killer cell induces programmed cell death, suggesting that genes that function during the telogen are related mainly to apoptosis.

### Signaling and transcription pathways during the hair cycle

**Fig. 5:**
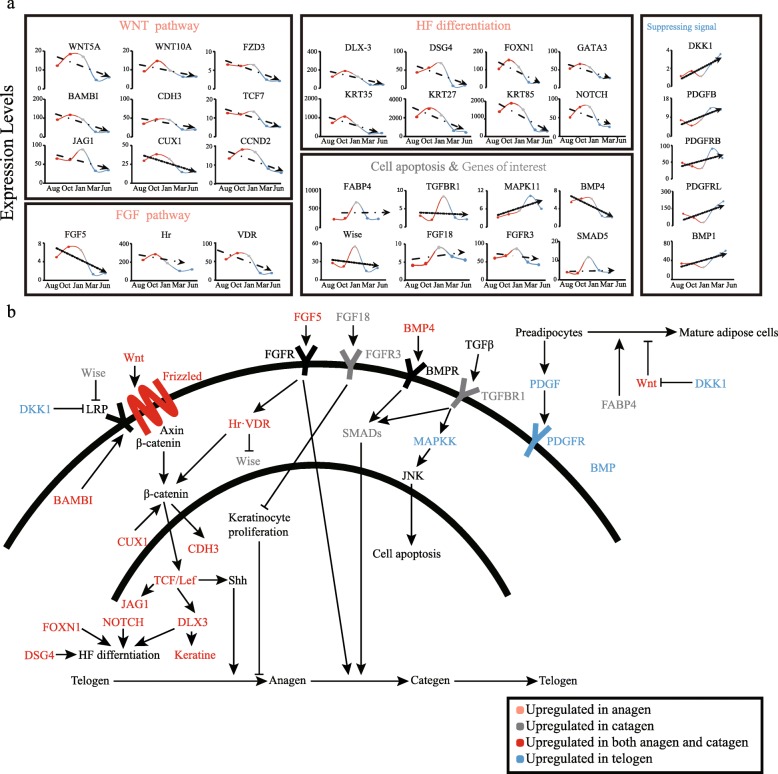
Expression patterns and regulatory networks of related signaling factors during hair cycle. **a** Changes in expression levels of key DEGs; **b** Interaction of key DEGs during the hair cycle. A line indicates interaction between the two genes. For each directional solid line, the beginning of the line is the upstream gene, the end is the downstream gene. An arrow indicates that the upstream gene activates the downstream gene, and a blocked line indicates that the upstream gene inhibits the downstream gene

After functional analysis, we investigated in more detail changes in the DEGs involved in the biological processes referred to above, in order to provide insights into the relationships among the DEGs during the hair cycle (Fig. 5). Some of the DEGs may serve as regulatory signals for proliferation and differentiation of skin and HFs cells during development and homeostasis, or play an important part in HFs stem cell activation. Differentiation and cycling may involve several key signaling pathways, which include Wnt, Bmp, Shh and Notch. The regulation of mammalian hair cycling is complex, requiring the convergence of many signaling pathways.

Interestingly, we found that Wnt, Notch, and FGF pathway members and the transcription factors that regulate keratin and HFs differentiation are more highly expressed in anagen compared to catagen and telogen (Fig. 5a). In the Wnt signaling pathway, expression of Wnt family members leads to accumulation of β-catenin mediated through the receptors of frizzled protein, which promote cooperation with *TCF/Lef* to induce genes encoding factors, such as homeobox gene family members and Shh, that trigger the hair cycle to transition from telogen (resting) to anagen (growth) [15]. Many key transcription factors are involved in the process described above (Fig. 5b). *BAMBI* interacts with the Wnt receptor *Fzd* and the co-receptor *LRP6* and promotes Wnt-induced cell cycle progression and proliferation [16]. *CUX1* stimulates *TCF/β-catenin* transcriptional activity [17]. *JAG1* and *CDH3*, as target genes of the WNT signaling pathway, are key factors in the transition of hair from early anagen to late anagen [18]. HFs differentiation is characterized by development of all the compartments of the HFs, and different signal molecules play specific roles in this process. *FoxN1* and *Notch* gene products play an important role in regulating HFs differentiation with Wnt5a mediators [19]. *DLX-3* acts as a transcription factor downstream of Wnt signaling; it controls the expression of keratin in hair development, and along with *DSG4* it controls hair follicle cell differentiation [20, 21]. *VDR* and *Hr* are two gene products essential for normal hair cycling in mammals [22]; they suppress *Wise* expression in vivo and allow hair cycle progression [23].

Among the genes highly expressed during catagen, we found a series of transcription factors that regulate the transition of the hair cycle from anagen to catagen. *Fgf18* expression in hair follicles is strictly associated with the catagen and telogen phases of the hair growth cycle; it will immediately inhibit the proliferation of stromal cells and strongly suppress the growth of hair follicles [24]. Wise inhibits the Wnt signaling pathways in the course of the hair cycle by binding to *LRP* receptors [25]. *BMP4* can interact with *Fgf18* to inhibit the activation of hair follicle stem cells. *Fgf5* and its receptor can promote the transition from anagen to catagen [26]. We found that *BMP4* and *Fgf5* are highly expressed in both anagen and catagen stages; this may indicate that they have a more prolonged effect on the hair cycle than other transcription factors and they may have some unknown effect during anagen. In the telogen, we found that transcription factors related to hair follicles growth inhibition are activated, such as *DKK1, PDGF* and *BMPs*.

### Co-expression network construction and analysis

**Fig. 6:**
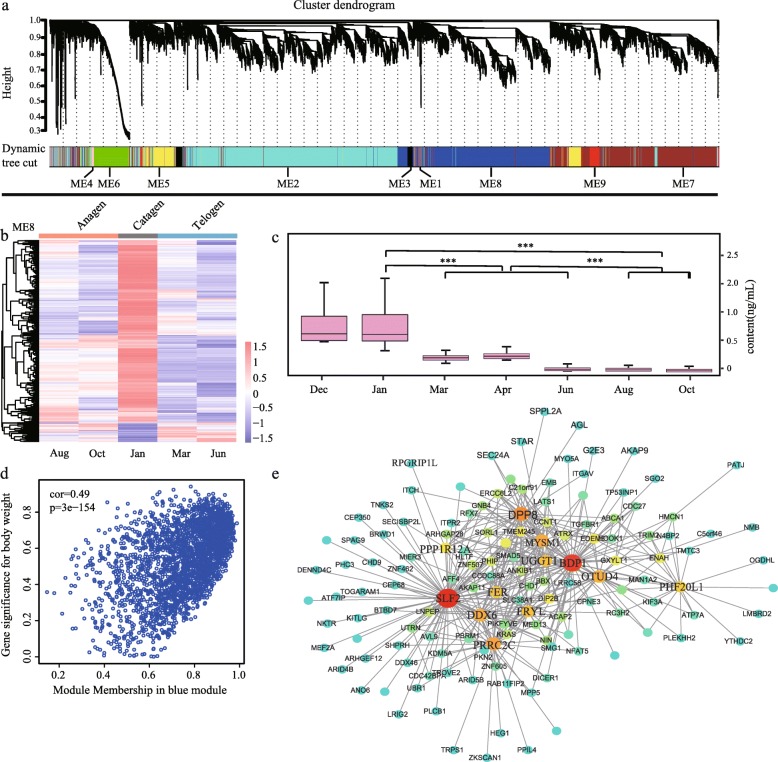
Global co-expression networks and gene modules. **a** Dendrogram from gene co-expression network analysis. Each module of co-expressed genes was assigned a color and number (ME1 to ME9); **b** Left: heat map of genes in ME8 showing the spatio-temporal expression pattern after hierarchical clustering. The expression values for each gene are arranged in the heat map, ordered first by anagen, then by catagen and last by telogen; **c** Changes in testosterone contents levels in different months. *P* values were calculated by unpaired t-test. *** indicate P value < 0.005; **d** Correlation between module membership and gene significance in the Blue module; **e** The top 40 genes defined by the highest intramodular connectivity and their associated genes are shown in the network view. Nodes and size are based on the extent of intramodular connectivity. Larger nodes are highly connected within a module and have greater ‘hubness’.

To extract additional biological information embedded in the transcriptome data set, we used the WGCNA software package in R to identify modules of co-expressed genes, and a total of 10 modules were obtained (Fig. 6a). Among these 10 modules, the grey module represents the genes that cannot be assigned to any module, and the number of genes contained in each module varies widely. The black module (ME3) contains the fewest, only 49 genes, while the blue module (ME8) contains the most, with 1015 genes.

We measured the in vivo hormone contents from different samples (Additional file 6: Figure S3) and correlated them with the module (Additional file 7: Figure S4). Our results show that the prolactin (PRL) content gradually increased from Dec to Mar, and began to rise after it fell to a trough in Apr. After Jun, the PRL content began to decline again until Dec; Testosterone (T) content is clearly divided into three levels, the highest in catagen, the lower in telogen, and the lowest in anagen; The Estradiol (E2) content during the anagen was significantly lower than the other periods and reached its lowest value in Oct; The trend change of thyroid stimulating hormone (TSH) content is basically consistent with T hormone; The change trend of melatonin (MT) content is exactly opposite to the change trend of sunshine duration. When the sunshine duration decreases, the MT content increases, and when the sunshine duration increases, the MT content decreases; Both insulin-like growth factor 2 (IGF2) and growth hormone (GH) contents increased significantly in catagen, while the telogen and anagen contents did not change significantly; The content of IGF1 has been stabilized without significant change.

In the Correlation analysis of hormones and modules, we found that the correlation between module M8 and testosterone content was 0.71 (*p* value 0.003), indicating that the genes in M8 may be associated with testosterone. ME8 module consisted of 1015 genes, which had the highest expression levels in catagen and low expression in the other two periods (Fig. 6b). The gene expression trend of ME8 was consistent with the change trend of testosterone (T) content (Fig. 6c). The genes with the highest degree of connectivity in the module and had high gene significance measures with the hormone are called hub genes and are considered to have important functions within the module (Fig. 6d). In the genetic interaction network, we found that ME8 hub genes included genes *SLF2, BOP1, DPP8* (Fig. 6e), explain that they are the pivotal genes of the module.

## Discussion

Animal coats play an important role in adaptation to the external environment. Non-hibernating land mammals can adapt to their surrounding conditions through seasonal changes to their coats [27]. Mammalian hair is produced by the proliferation and differentiation of hair follicle cells [28]. The hair follicle, which is the only life-sustaining organ unique to mammals [29], plays an important role in mammalian adaptation to environmental changes. Many signals and hormones involved in the hair follicles cycle have been studied, for example, Wnt, Shh signaling pathway; *Tcf3/4, Lhx2, p63, Dlx3, Lef1* and other transcription factors [4]; Among hormones are Estradiol, melatonin, prolactin, etc. Although many such pathways and transcription factors are involved in the formation and growth of the yak hair follicle, the exact nature, timing, and interaction of these induction and regulatory signals are still difficult to determine. The yak is a rare large livestock animal that lives in alpine and hypoxic environments, and the dense hair helps the yak extremely resistant to the cold environment in winter, but in contrast, this prevent them from sufficiently emitting heat in summer or hotter area; this requires yak to respond very sensitively to temperature differences in different seasons. We selected yak as a model species for our study of hair development, because its hair follicles are in a dynamic relationship with environmental changes, resulting in marked periodic changes in the structure of the hair follicles, and the developmental regulation of this process is very rapid [8]. Sequencing and analyzing yak skin samples during different seasons might help explain how yak have adapted to the harsh environment of the alpine region by regulating the hair follicle cycle.

In the hierarchical clustering plot, the 15 samples could be grouped into three hair follicles cycle phases, and we found that one sample from Jun was clustered together with the Mar samples. This phenomenon indicates, on the one hand, that the individual differences between the sampled yaks were relatively large, and on the other hand, that the gene expression patterns of the samples from March and June are very similar. In addition, we observed that the clustering of the anagen samples was closer to that of the catagen samples than to that of the telogen samples. This result indicated that the development status of the catagen, the dynamic transition between anagen and telogen, was closer to that of the anagen. In the study of the expression patterns of DEGs at three developmental stages during the hair cycle, we found the largest number of upregulated and downregulated DEGs during the telogen. We speculate that the developmental transitions from telogen to anagen and catagen to telogen were highly dynamic.

After we thoroughly investigated the DEGs at different developmental stages, we found some GO and KEGG pathways that are highly related to the hair cycle, as well as transcription factors involved in regulating the hair cycle. The functions of genes highly expressed in the anagen and catagen are related to signal induction and hair generation, such as Wnt, Shh signaling pathway, synthesis of intermediate filament and keratin filament, the pluripotency of stem cells. In the process of hair follicle development, the Wnt and Hedgehog signaling pathways are key to stimulation of hair follicle morphogenesis, the transition from telogen to anagen occurs when one or two dormant stem cells at the base of the telogen follicle near the dermal papilla are activated by the interplay between Wnt, Shh and Notch factors to produce a new hair shaft [15, 30, 31]. activation of the pluripotency of stem cells plays an essential role during hair follicle formation [32]. Intermediate filament and keratin filament are essential elements in the process of hair formation [33], and we found a large number of keratin that is highly expressed during the anagen of hair cycle. The function of catagen highly expressed genes is mainly related to the inhibition of hair follicle development, indicating that the main function of these genes is to promote the transformation of hair follicles from anagen to telogen. The telogen can be divided into refractory (early period) and competent (end period) stages. In the early to mid telogen, growth-suppressing signals such as *DKK1* are in play, followed by active repression of quiescence mediators, mainly *PDGF* and *BMPs*, during late/competent telogen [34]. In summary, each signaling pathway and transcription factors together form a complex signal transmission network and participate in the hair follicles cycle.

According to previous reports, Among the eight hormones that affect hair follicle development, E2, PRL and T mainly inhibit hair follicle development. E2 can induce hair follicles in the anagen to enter the catagen early, and then keeps the hair follicles at telogen [35]. PRL is an important hormone that controls hair follicle activity and villus shedding. When its content reaches a high peak, villus begins to grow; as the hormone content decreases, villus growth accelerates; when the content increases again, villus stops growing [36]. GH, IGF1, IGF2 and MT mainly promote hair follicle development. The content of GH reached a high level in summer, and the hair began to grow rapidly, and then in winter, hormone levels to the lowest, hair stops growing [37]. IGF1 promotes the growth of hair, but has little effect on the cyclical changes of hair growth [38]. The content of MT changes with the duration of sunshine, the secretion of MT decrease during long sunshine and increases during short sunshine [39]. while the effect of TSH hormone is still unclear. Our results show that most of the hormones are consistent with previous studies. The E2 content of yak in the anagen was significantly lower than catagen and telogen, when the yak hair follicle cycle changes from the anagen to catagen, the content of E2 begins to increase. The content of PRL start to increase during telogen, and decreases gradually during the anagen, which may be to prepare for villus development in the anagen. The content of MT decreased with the increase of sunshine duration. In short, these hormones show significant cyclic changes and may interact with some genes and transcription factors in the regulation of the yak hair follicle cycle. Among these hormones, we focused on T. On the one hand, T is highly correlated with the module gene, on the other hand, T plays an important role in the hair cycle. In previous studies of humans, T is converted to di-hydro-testosterone (DHT) by 5α-reductase, then binds to the androgen receptor in the hair follicle target cells, inhibiting the growth and development of the dermal papilla, interfering with the growth and metabolism of hair follicle cells, and advancing the hair into the catagen [18]. During the transition from anagen to catagen, the T content in yak blood increased significantly. We speculate that T plays an important role in promoting the yak hair follicle cycle into the catagen. Genetic interaction network can help us identify interactions with genes that are highly related to hormones. We speculate that the Hub genes in ME8 are probably play an important role in the process of testosterone affecting the hair cycle.

## Conclusions

In conclusion, this study used high-quality RNA-seq data to analyze the dynamic expression of the yak transcriptome during the hair cycle. Through cluster analysis, we found that the yak hair cycle can be divided into three periods with their own specific DEGs. The results of differential expression analysis suggested that the transitions from telogen to anagen and catagen to telogen were highly dynamic. GO and KEGG enrichment analysis suggested that the functions of genes that were highly expressed during anagen were related to signal induction and hair generation. We describe the regulatory network of related signaling factors active at different stages during the hair cycle based on the dynamic expression of DEGs. We performed co-expression network analysis to identify genes that play vital roles in the hair cycle and identified several key genes that may be involved in its hormonal regulation. These results lay a foundation for an increased understanding of the molecular mechanisms underlying the yak hair cycle and identification of new developmental regulators, which may promote our understanding of the mechanism of hair development in not only yak but also other mammals.

## Methods

### Sample collection

The sample collection work in this project was completed in the Gansu Key Laboratory of Yak Breeding Engineering. All yaks in this study are bred in a pasture owned by individual herders in Duolong Village, Tianzhu Tibetan Autonomous County, Wuwei City, Gansu Province. For transcriptome sequencing, we selected the healthy female white yaks around 2 ~ 3 years old under uniform growth environment and feeding conditions. According to the periodical variation of yak hair and the annual temperature changes in Tianzhu region, which were recorded by the Tianzhu Regional Meteorological Bureau (Figure S1). We conducted a total of fifteen sample collections from fifteen yaks at five time points of January, March, June, August and October, covering the three phases of the yak hair follicle development.

The blood samples and skin samples were collected at the same time. The blood samples were collected from the jugular and without the addition of anticoagulants, the blood was centrifuged at 3500 rpm for 10 min, and the supernatant was taken for determination of serum hormone levels. The skin samples were collected from the scapulae region using a medical skin sampler. We used PBS to remove residual blood and stains, then absorbed the surface liquid with absorbent paper and store in liquid nitrogen.

### Physiological data measurements

The hormone levels were measured for 7 points, including January, March, April, August, October and December, respectively, each time with 22 ~ 25 samples (Additional file 6: Figure S3). Determination of serum hormone levels: 5 mL of blood was collected from the jugular of the yak, and placed in a vacuum blood collection tube without added anticoagulant, and serum was prepared by centrifugation at 3500 rpm for 10 min at 4 °C. Serum hormone levels were determined by enzyme-linked immunosorbent assay (ELISA). We followed the instructions for each test kit to determine the serum concentrations of eight hormones: Estradiol (E2), testosterone (T), thyroid stimulating hormone (TSH), prolactin (PRL), melatonin (MT), growth hormone (GH), insulin-like growth factor 1 (IGF1), insulin-like growth factor 2 (IGF2), using a DR-200BS enzyme standard analyzer.

### Next-generation sequencing

RNA was extracted from skin at five developmental stages (3 replicates per stage) using a mirVana™ miRNA Isolation Kit (Ambion-1561) following the manufacturer’s protocol. RNA integrity was evaluated using an Agilent 2100 Bioanalyzer (Agilent Technologies, Santa Clara, CA, USA). Samples with RNA Integrity Number (RIN) ≥ 7 were subjected to subsequent analysis. The libraries were constructed using a TruSeq Stranded mRNA LTSample Prep Kit (Illumina, San Diego, CA, USA) according to the manufacturer’s instructions. Then these libraries were sequenced on the Illumina sequencing platform (HiSeq 2500) and 150 bp paired-end reads were generated.

### Mapping and assembly of RNA-seq data

Before mapping, raw data were filtered in the following steps: a. Removal of the joint sequence at both ends of Reads; b. Elimination of unidentified bases (N) from one of the Reads in Paired-end sequencing, which accounted for more than 5% of the total length of the Reads; c. Removal of Paired-Reads with a Read containing > 65% low quality bases; d. Trimming of each Read, removal of consecutive low-quality bases from both ends. The remaining reads were defined as “clean reads” and used for subsequent bioinformatics analyses. Quality control on the clean data was performed by producing a base composition chart and quality distribution chart using FastQC V0.11.8 (http://www.bioinformatics.babraham.ac.uk/projects/fastqc/). We used HiSat2 [40] to map clean reads to the yak genomic sequences (GenBank accession number *AGSK01000000*). Prior to this, exons and splice sites were extracted from the genome annotation GTF files and used to index the genomes. The mapping statistics and the coverage of the gene body by the RNA-seq reads were calculated using Samtools V1.9 [41]. Then we applied a reference-based transcript assembler, StringTie V22 [42], to assemble the transcripts for each sample.

### Differential gene expression analysis

Calculating gene expression using FPKM can eliminate the influence of gene length and sequencing data quality on apparent gene expression. The parameter FPKM (Fragments per kilobase of transcript per million mapped reads) was estimated by running StringTie with the -eB options. Ballgown V2.12.0 was applied to quantify gene expression levels [43]. Read count information was extracted from the above transcript abundance files by prepDE.py for subsequent differential expression analysis. We used the R statistical package DESeq2 V1.20.0 from the Bioconductor repository to test for differential expression among the samples from different stages [44]. The screening criteria used were: (1) The standard deviation of FPKM was greater than 1 in at least one sample in order to remove low-abundance transcripts, (2) log2 (FPKMtime1/FPKMtime2) was > 1 or < − 1, (3) *P* value of the significance test (FDR) < 0.05.

### Classification and annotation of DEGs

We used MEV V4.9.0 for sequence cluster analysis [45]; the differentially expressed genes were grouped into nine clusters according to their FPKM trend across different stages of the hair cycle. Fisher’s exact test and multiple comparison tests were used to calculate the significance levels of profiles [46]. Functional enrichment analysis was performed on the different classes of DEGs in the nine K-means clusters.

The purpose of Gene Ontology (GO) analysis is to elucidate the biological implications of unique genes appearing in significant or representative profiles [47]. For each GO category, we used a chi-square test and Fisher’s exact test to identify the significant GO categories and FDR was used to correct the *p*-values by means of the ‘p.adjust’ function. The threshold of significance for GO functional classification was defined by p-value < 0.05 and FDR < 0.05.

KEGG Pathway analysis was applied to identify significant pathways among the differentially expressed genes. A chi-square test and Fisher’s exact test were used to find significantly enriched pathways with a threshold of significance set at p-value < 0.05 and FDR < 0.05.

Signaling and transcription pathway analysis was conducted to provide insight into the relationship of DEGs based on potential interactions. In this analysis, nodes represent genes and arrows represent interactions between genes, including activation or inhibition.

### Construction of co-expression modules

The R package WGCNA V1.64.1 (weighted gene co-expression network analysis) was used to identify modules of highly correlated genes based on the FPKM data [48]. An independent signed networks was constructed using the FPKM data for the filtered genes acquired through Ballgown. We selected a soft threshold power of 9 to establish an adjacency matrix according to the degree of connectivity so that our gene distribution conformed to the scale-free network. The TOM similarity algorithm was used to convert the resulting adjacency matrix into a topological overlap (TO) matrix. Average hierarchical clustering using the ‘hclust’ function was applied to group genes according to TO similarity. One-step network modules were constructed using a dynamic tree cut algorithm with a minimum cluster size of 30, a merging threshold function of 0.30 and a medium sensitivity (deepSplit = 2) to cluster splitting. Each module was summarized by the module eigengene (ME).

To relate the physiological measurements to the network, we correlated the module eigengenes with the hormone content data and calculated Gene Significance (GS) as the absolute value of the correlation between gene expression and physiology across the time series. Module membership (MM) is used to measure the importance of a gene in a module. It is generally believed that the higher the absolute values of MM and GS for genes, the more important those genes are in the module and the greater the correlation with, in this case, hormone contents. When screening for hub genes in the module, we therefore set thresholds for GS and MM, and identified genes with GS > 0.7 and MM > 0.9 as hub genes. Cytoscape V3.6.1 software was used to visualize hub genes in the module [49].

## Supplementary information

**Additional file 1 Figure S1.** The changes of yak habitat temperature.

**Additional file 2 Table S1.** Characteristics of the reads from 12 yak skin transcriptomes.

**Additional file 3 Figure S2.** Venn diagram pf the DEGs of anagen, catagen and telogen.

**Additional file 4 Table S2.** GO terms enriched by the DEGs in the nine expression patterns.

**Additional file 5 Table S3.** KEGG pathways enriched by the DEGs in the nine expression patterns.

**Additional file 6 Figure S3.** Changes in eight hormones contents levels in different months. *P* values were calculated by unpaired t-test. *** indicate P value < 0.005, ** indicate P value < 0.05, NS indicate not significant.

**Additional file 7 Figure S4.** Correlation analysis of hormones and modules.

## Data Availability

All the sequencing data are available at the NCBI under accession number PRJNA550233 (https://www.ncbi.nlm.nih.gov/bioproject/PRJNA550233). At the same time, we also added the SRA accession numbers corresponding to each sample (Additional file 2: Table S1).

## Abbreviations

HFs: Hair follicle
DEGs: Differentially expressed genes
FPKM: Fragments per kilobase of transcript per million mapped reads
GO: Gene ontology
KEGG: Kyto encyclopaedia of genes and genomes
MTOR: Mechanistic target of rapamycin
Bmp: Bone morphogenesis protein
Shh: Sonic Hedgehog
FGF: Fibroblast growth factor
Fzd: Frizzled protein
DKK: Dickkopf
PDGF: Platelet-derived growth factor
DHT: Di-hydro-testosterone
