# Supplementary figures and images for: The seasonal development dynamics of the yak hair cycle transcriptome
Source: BMC Genomics. 2020 May 11;21:355. doi: 10.1186/s12864-020-6725-7 (PMC7216598; doi:10.1186/s12864-020-6725-7)

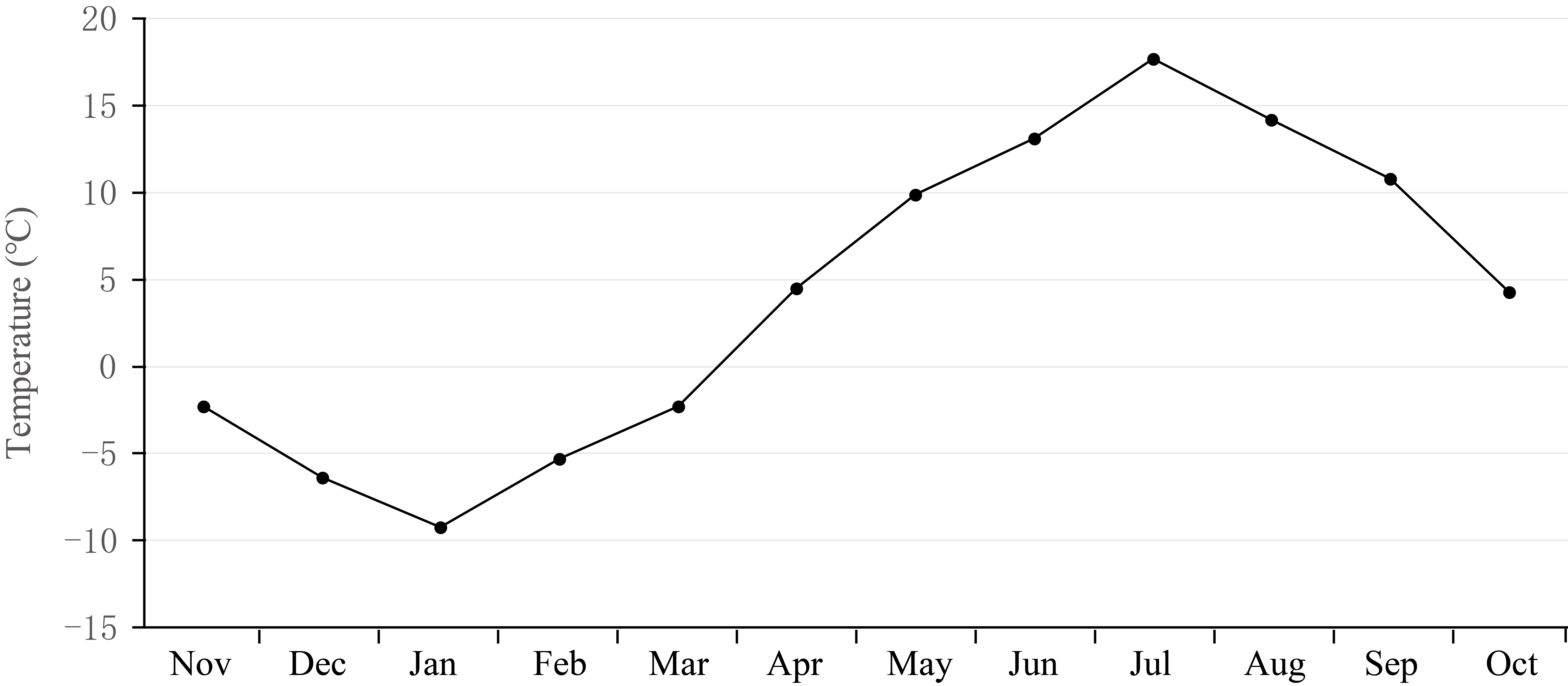

Supplement: Supplementary file 1 — Additional file 1 Figure S1. The changes of yak habitat temperature. [file 12864_2020_6725_MOESM1_ESM.png]

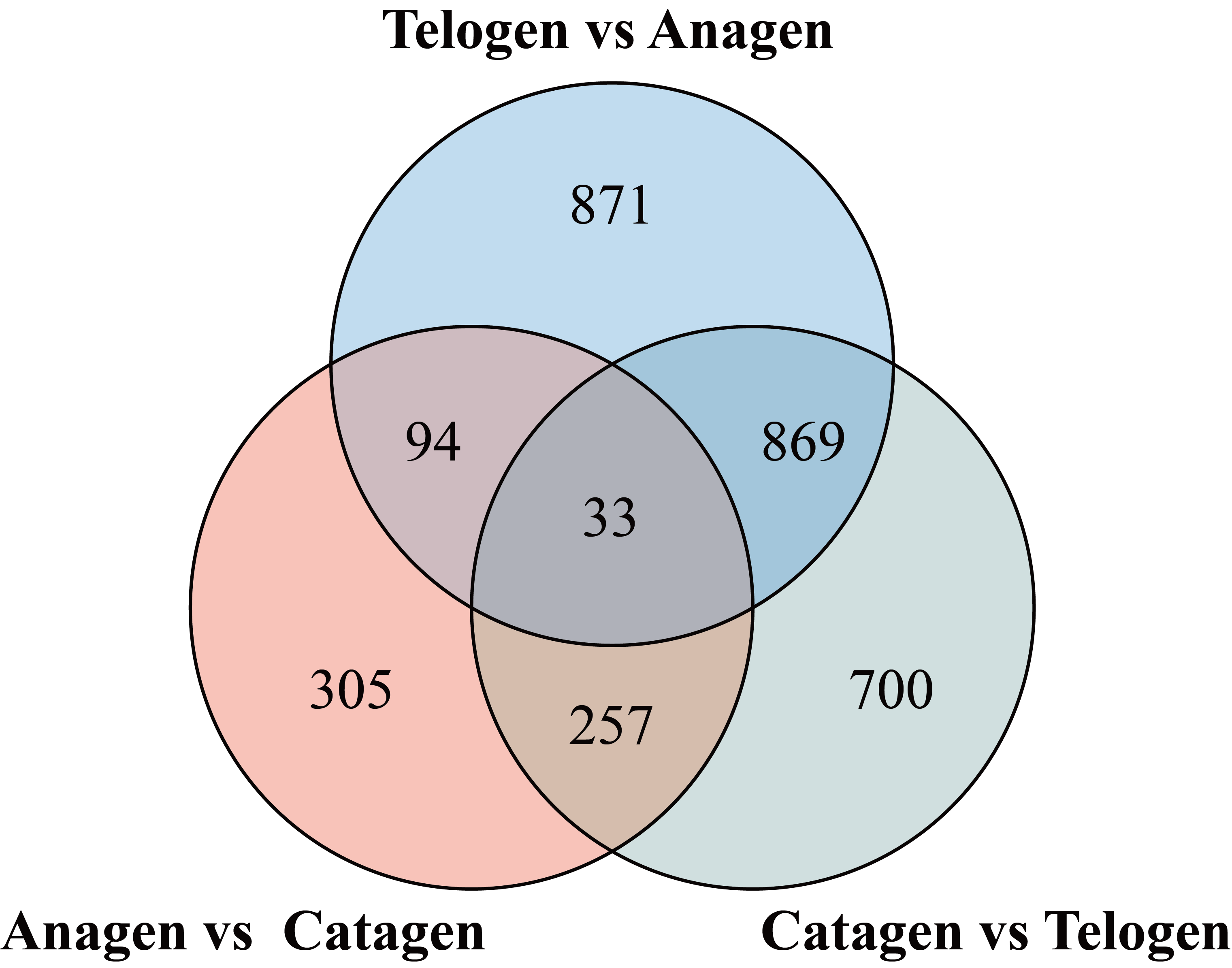

Supplement: Supplementary file 3 — Additional file 3 Figure S2. Venn diagram pf the DEGs of anagen, catagen and telogen. [file 12864_2020_6725_MOESM3_ESM.png]

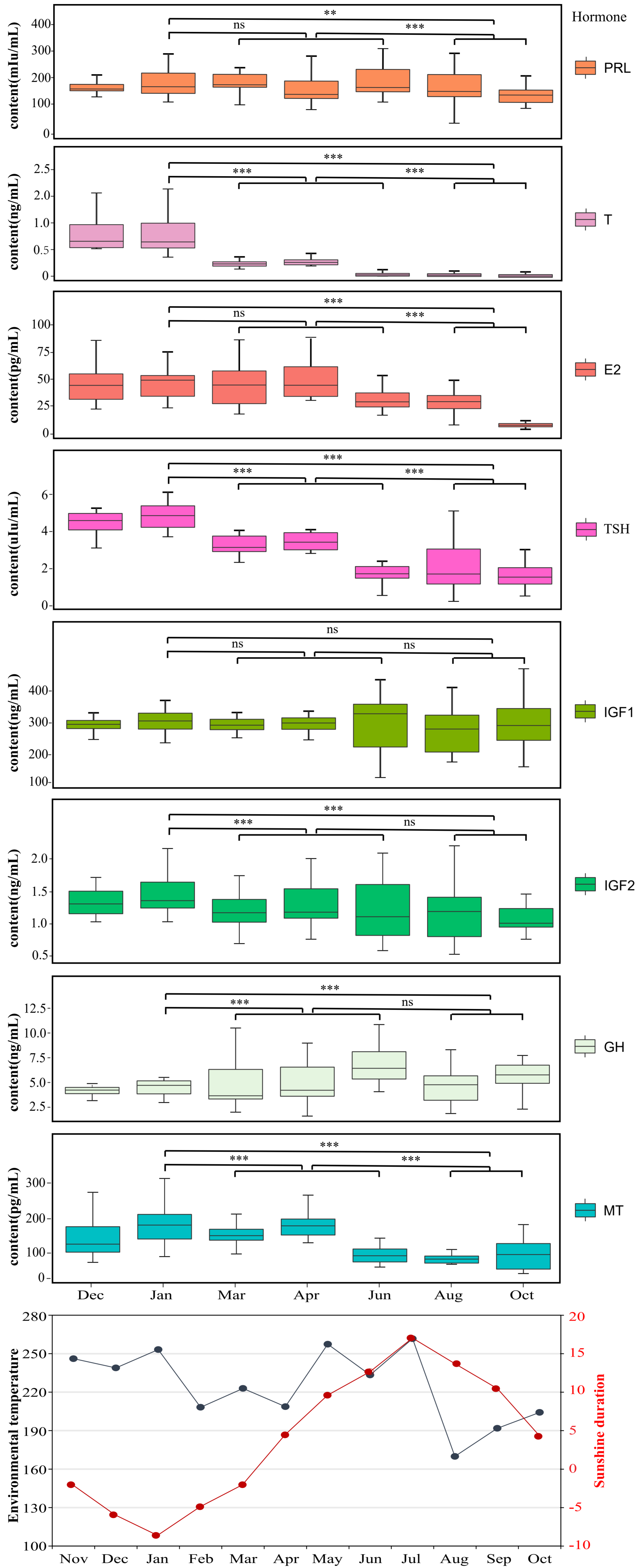

Supplement: Supplementary file 6 — Additional file 6 Figure S3. Changes in eight hormones contents levels in different months. P values were calculated by unpaired t-test. *** indicate P value < 0.005, ** indicate P value < 0.05, NS indicate not significant. [file 12864_2020_6725_MOESM6_ESM.pdf]

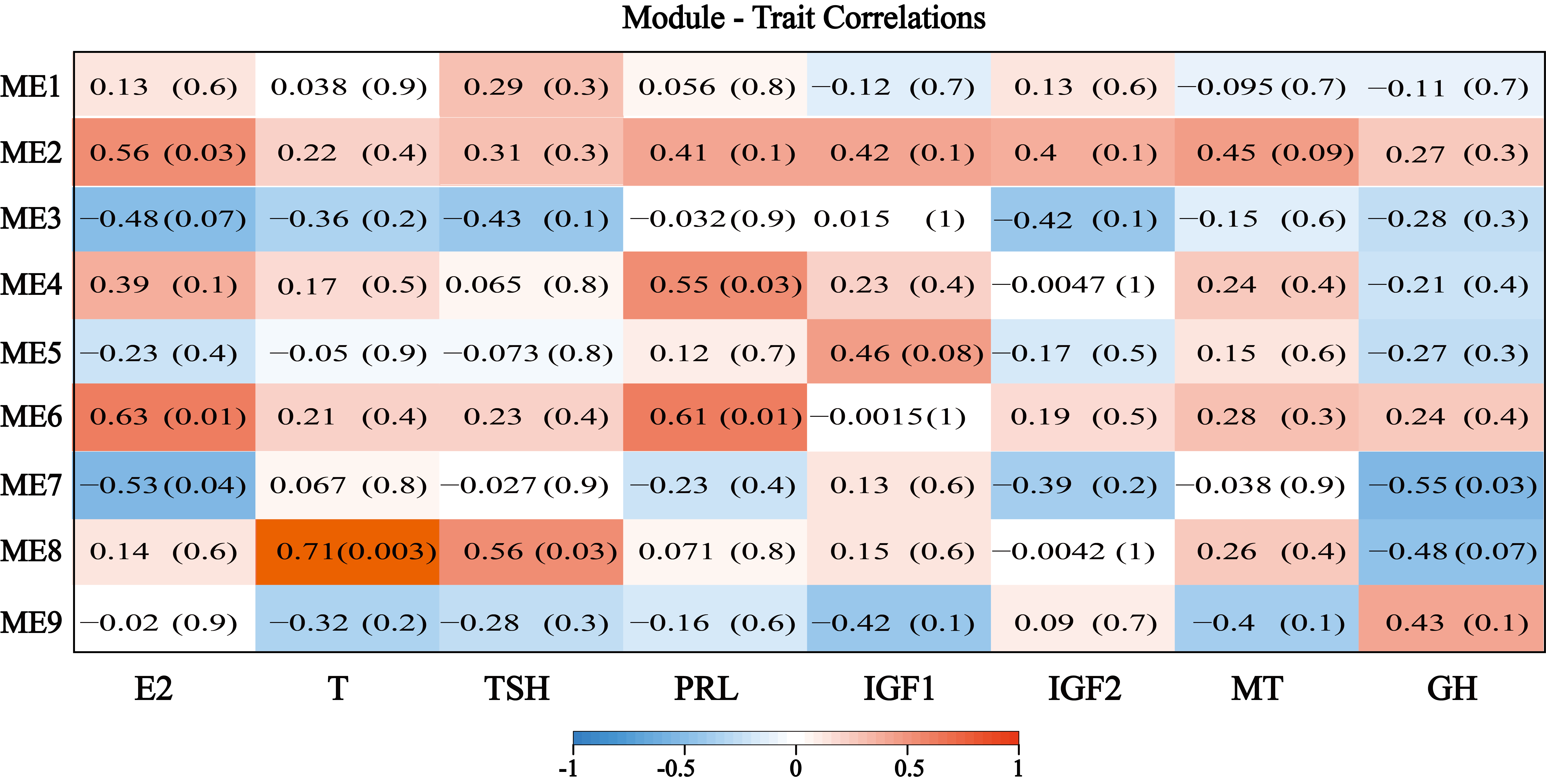

Supplement: Supplementary file 7 — Additional file 7 Figure S4. Correlation analysis of hormones and modules. [file 12864_2020_6725_MOESM7_ESM.png]
