# Supplementary material for: The seasonal development dynamics of the yak hair cycle transcriptome
Source: BMC Genomics. 2020 May 11;21:355. doi: 10.1186/s12864-020-6725-7 (PMC7216598; doi:10.1186/s12864-020-6725-7)
Supplement: Supplementary file 2 — Additional file 2 Table S1. Characteristics of the reads from 12 yak skin transcriptomes. [file 12864_2020_6725_MOESM2_ESM.docx]

**Additional file 2: Table S1. Characteristics of the reads from 12 yak skin transcriptomes**

| Sample | Total bases | Raw reads pairs | Clean reads pairs | Total mapped reads | Accession numbers |
| --- | --- | --- | --- | --- | --- |
| Aug1 | 9,910,080,212‬ | 33,682,668 | 33,532,123 | 64,567,762(91.62%) | SRR10059232 |
| Aug2 | 9,055,073,008‬ | 30,785,529 | 30,638,716 | 59,488,417(92.35%) | SRR10059244 |
| Aug3 | 9,273,365,710‬ | 31,515,448 | 31,364,765 | 60,338,839(91.85%) | SRR10059243 |
| Oct1 | 7,109,221,161 | 24,027,197 | 23,939,096 | 46,446,749(92.63%) | SRR10059242 |
| Oct2 | 7,100,100,223 | 23,956,540 | 23,877,759 | 46,473,049(92.86%) | SRR10059241 |
| Oct3 | 6,976,201,191‬ | 23,562,320 | 23,484,544 | 45,494,144(92.41%) | SRR10059240 |
| Jan1 | 6,083,262,614 | 21,302,916 | 20,824,764 | 40,085,550(92.06%) | SRR10059246 |
| Jan2 | 6,072,370,013 | 21,311,614 | 20,816,802 | 40,442,527(92.68%) | SRR10059245 |
| Jan3 | 5,916,412,167‬ | 20,809,764 | 20,311,226 | 39,895,301(93.03%) | SRR10059239 |
| Mar1 | 7,134,874,376‬ | 24, 296,646 | 24,182,290 | 46,659,631(92.46%) | SRR10059238 |
| Mar2 | 7,154,489,297‬ | 24,364,619 | 24,259,851 | 46,231,990(91.10%) | SRR10059237 |
| Mar3 | 7,081,227,910 | 24,089,930 | 23,981,258 | 46,382,421(92.02%) | SRR10059236 |
| Jun1 | 7,210,705,417‬ | 24,549,950 | 24,446,407 | 46,101,749(90.61%) | SRR10059235 |
| Jun2 | 7,208,432,905‬ | 24,524,389 | 24,405,793 | 47,524,276(93.17%) | SRR10059234 |
| Jun3 | 7,179,766,111‬ | 24,455,699 | 24,330,734 | 46,916,554(92.35%) | SRR10059233 |
